# An optimally evolved connective ratio of neural networks that maximizes the occurrence of synchronized bursting behavior

**DOI:** 10.1186/1752-0509-6-23

**Published:** 2012-03-31

**Authors:** Chao-Yi Dong, Kwang-Hyun Cho

**Affiliations:** 1 Department of Automatic Control, Inner Mongolia University of Technology, Huhhot 010080, People's Republic of China; 2 Department of Bio and Brain Engineering, Korea Advanced Institute of Science and Technology (KAIST), Daejeon 305-701, Republic of Korea

## Abstract

**Background:**

Synchronized bursting activity (SBA) is a remarkable dynamical behavior in both *ex vivo *and *in vivo *neural networks. Investigations of the underlying structural characteristics associated with SBA are crucial to understanding the system-level regulatory mechanism of neural network behaviors.

**Results:**

In this study, artificial pulsed neural networks were established using spike response models to capture fundamental dynamics of large scale *ex vivo *cortical networks. Network simulations with synaptic parameter perturbations showed the following two findings. (i) In a network with an excitatory ratio (ER) of 80-90%, its connective ratio (CR) was within a range of 10-30% when the occurrence of SBA reached the highest expectation. This result was consistent with the experimental observation in *ex vivo *neuronal networks, which were reported to possess a matured inhibitory synaptic ratio of 10-20% and a CR of 10-30%. (ii) No SBA occurred when a network does not contain any all-positive-interaction feedback loop (APFL) motif. In a neural network containing APFLs, the number of APFLs presented an optimal range corresponding to the maximal occurrence of SBA, which was very similar to the optimal CR.

**Conclusions:**

In a neural network, the evolutionarily selected CR (10-30%) optimizes the occurrence of SBA, and APFL serves a pivotal network motif required to maximize the occurrence of SBA.

## Background

In the brain development, neurons are assembled together via numerous synapses to build up complicated neuronal networks performing specific behaviors, such as transient or sporadic activity, synchronized bursting activity (SBA), and hyper-excitable activity. One of the most prominent behaviors in cortical networks is the synchronized bursting spikes occurring in the brain development and maturation [1-3]. The behavior is not only found in *ex vivo *cultured cortical networks [4] but also in the brain regions of several *in vivo *animal models like visual cortex [5], hippocampus [6], and auditory neocortex [7]. In particular, under *in vivo *conditions, SBA is considered highly related to a variety of crucial biophysical functions, such as attentional selection [8-10], cognitive motor processes [11], visual pattern recognition [12], auditory object perception [13].

Although SBA is an unique phenomenon in neuronal networks, characteristics of the neural networks causing SBA remain unknown, in contrast to the study on the function significance of the SBA [14]. Presently, large random *ex vivo *cortical networks are more appropriate experimental model systems in the studies on the universal mechanisms governing the formation and conservation of neural network activities. Experiments using *ex vivo *cultured neural networks have demonstrated that the adjustment of synaptic connections is highly correlated with the development of neuronal network behavior such as the evolution of spontaneous electrical activity [15,16]. In the matured phase of an *ex vivo *cultured neural network, each neuron builds up synaptic connections with 10-30% of other neurons within the neural network [17,18]. Another line of evidence has indicated that the electrical activity of neurons can directly affect the outgrowth of neurites, and such reconfiguration of neuronal networks in turn causes adaptive adjustment of the neuronal electrical activity [19]. This behavior-dependent regulatory mechanism precisely drives and controls networks to grow, prune, and finally converge to a proper connective ratio (CR) (10-30%).

According to the above described connectivity characteristics of *ex vivo *cultured neural networks, two interesting questions arise: why do such a matured neural network keep its CR within a fixed range (10-30%); and what biological significance and associated implications does this fixed CR have? To answer these intriguing questions, we hypothesize that the CR is associated with the facilitation of synchronized bursting network behaviors, since synaptic connections are always found correlated with network behaviors in *ex vivo *experiments. Spike response models [20-23] were used to construct randomly connected artificial pulsed neural networks. The connective weights between two neurons were randomly selected, and the CR of the networks was increased progressively to mimic the process of development of cultured neural networks. The correlation between network behavior and structure was investigated using simulations. Subjecting the simulations to parameter perturbations revealed that, for a network with an excitatory ratio (ER) at 80-90% (a realistic ratio for *ex vivo *networks), the CR of the network always lies in a range of 10-30% when the occurrence of SBA reaches its highest expectation. This value is consistent with the matured CR of *ex vivo *neuronal networks with the inhibitory synaptic ratio at 10-20% [24,25]. This result reveals that the networks are evolved to form such a CR for optimizing the occurrence of SBA rather than randomly connected.

This study also explored the relationship between the occurrence of SBA and the composition of network motifs in the neural networks [26,27]. We found that SBA can be found only in the networks containing an all-positive-interaction feedback loop (APFL) [2]. For networks containing APFLs, the number of APFLs also demonstrates an optimal range corresponding to the maximized occurrence of SBA, close to the CR. Thus, we infer that the APFL may serve a crucial network motif underlying to maximize the occurrence of SBA.

For a pilot study in real neural networks, we have employed the neural network of nematode worm *C. elegans *[28,29]. The nervous system of *C. elegans *consists of 302 neurons and the number of neurons is almost same for different individuals. Each neuron in *C. elegans*' nervous system has distinct properties in view of morphology, connectivity, and position, and therefore it can be labelled specifically. The neural network of *C. elegans *is highly clustered like regular lattices and also has small characteristic path lengths like random graphs. So, it is well represented by small-world networks [30,31]. We investigated the egg-laying circuit of *C. elegans *including 11 neurons or neuron classes to examine our major claims [32,33]. As a result, we found that the egg-laying circuit has 17.3% CR and 10.5% ER which lie within the aforementioned evolved ranges. We also found that three two-node APFLs included in this circuit contribute to inducing a much higher level of SBAs in contrast to the randomly connected networks with the same number of network nodes.

## Results

### The optimal CR at the maximal occurrence of SBA

To unravel the biological significance of the CR of matured neural networks (10-30%), we first investigated the relationships between CR, ER, and the occurrence of SBA. Spike response models (SRM) can be used to simulate random *ex vivo *cortical networks so that their fundamental dynamical properties can be modeled [34]. The detailed simulation protocols for the artificial pulsed neural networks constructed by SRM are introduced in the Methods section. The connective weights among neurons were randomly assigned in a certain range to obtain a result that was irrelevant to the specific value of connective weights. For each CR and ER, 1,000 randomly connected pulsed neural networks were constructed to generate various network behaviors which were then further classified into four major categories with respect to the proposed criteria in the section entitled "The typical behaviors of spike neural networks".

**Figure 1:**
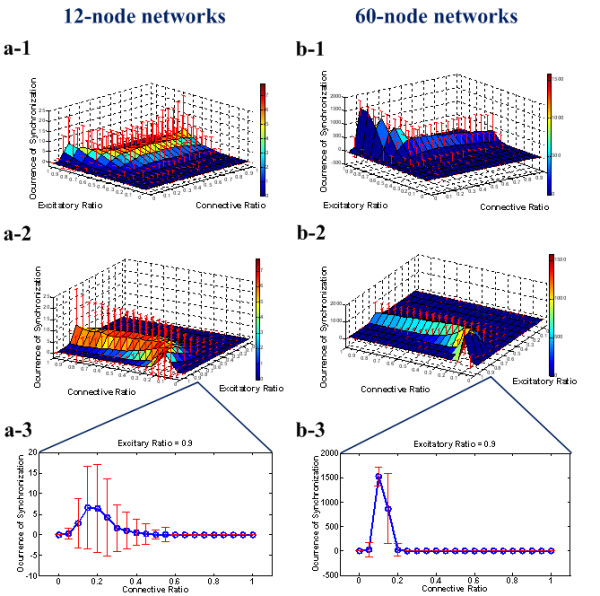
**The relationship between the occurrence of SBA and CR (or ER) for 12-node networks and 60-node networks**. **(a-1), (a-2)** The relationships among CR, ER, and the occurrence of SBA for 12-node networks from two different perspectives. The occurrence of SBA in 1,000 randomly connected networks was evaluated on each point of a lattice composed of CRs (0:0.05:1) and ERs (0:0.1:1). The surface points represent the mean value of the occurrence of SBA, while the upper and lower red bars show their standard deviations over 1,000 simulations. **(a-3) **The relationship between CR and the occurrence of SBA for 12-node networks when ER equals 0.9, which is consistent with the actual biological level. **(b-1), (b-2) **The relationship among CR, ER, and the occurrence of SBA for 60-node networks from two different perspectives. The occurrence of SBA in 1,000 randomly connected networks was evaluated on the same lattice as (a). The surface points represent the mean value of the occurrence of SBA, while the upper and lower red bars show their standard deviations over 1,000 simulations. **(b-3) **The relationship between CR and the occurrence of SBA for 60-node networks when ER equals 0.9 (the biological level).

**Figure 2:**
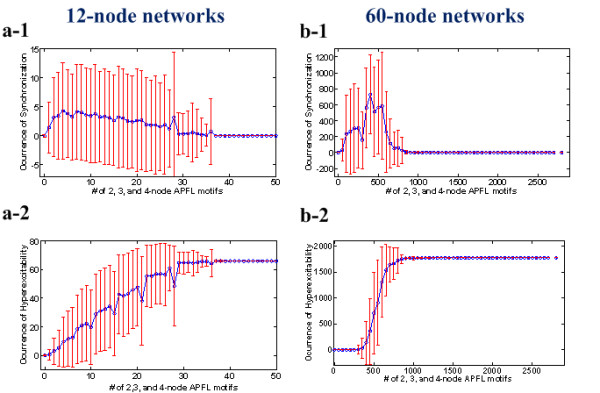
**The relationship between the total number of 2, 3, and 4-node APFL motifs and the occurrence of SBA or HEA for the 12-node networks and 60-node networks**. **(a-1)** The relationship between the total number of 2, 3, and 4-node APFL motifs and the occurrence of SBA for the 12-node pulsed neural networks. **(a-2)** The relationship between the total number of 2, 3, and 4-node APFL motifs and the occurrence of 2-channel HEA for the 12-node networks. **(b-1)** The relationship between the total number of 2, 3, and 4-node APFL motifs and the occurrence of SBA for the 60-node pulsed neural networks. **(b-2)** The relationship between the total number of 2, 3, 4-node APFL motifs and the occurrence of 2-channel HEA for the 60-node networks.

**Figure 3:**
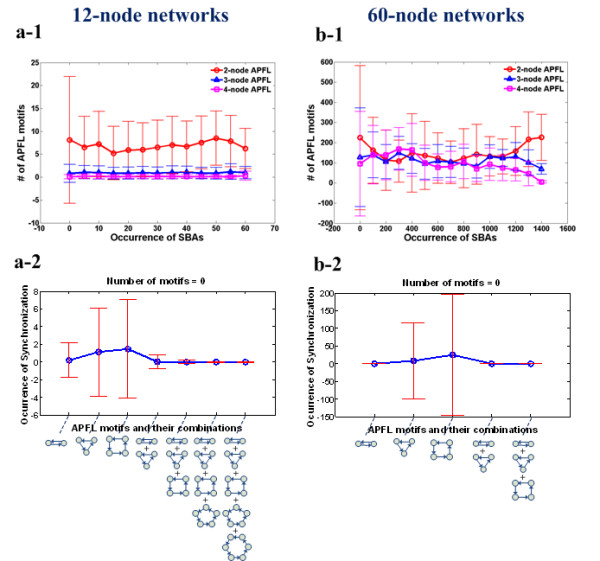
**The influence of 2, 3, or 4-node APFL motifs on inducing SBA. (a-1) **The number of 2, 3, or 4-node APFL motifs with respect to the level of SBA (12-node networks). **(a-2) **The occurrence of SBA along with the absence of particular APFL motifs or combinations of motifs in 12-node networks. **(b-1) **The number of 2, 3, or 4-node APFL motifs with respect to the level of SBA (60-node networks). **(b-2) **The occurrence of SBA along with the absence of particular APFL motifs or their combinations in 60-node networks.

The SBA properties of networks were investigated at two different scales: small networks with 12 nodes and large networks with 60 nodes. We recorded and calculated the expectation and standard deviation of SBA occurrence over the 1,000 networks (with a stereotyped CR and ER). Figure 1 shows the relationships among CR, ER, and the occurrence of SBA in the 12-node networks (Figure 1, a-1, a-2, a-3) and the 60-node networks (Figure 1, b-1, b-2, b-3). The mean and standard deviation of the occurrence of SBA were calculated over the 1,000 spike neural networks in each possible combination of CR and ER. The interesting profiles concerning the relationship between CR and the occurrence of SBA are found when the ER was equal to 0.9, which coincides with the experimental observation in cultured neural networks [25]. Figures 1 a-3 and b-3 show an optimal CR of 15% in the 12-node networks and 10% in the 60-node networks, respectively. This relation between CR and SBA suggests that the direction of evolutionary selection of CRs (not only in artificial pulse neural networks but also in *in vitro *neural networks) is to maximize the possibility for synchronized bursting behavior by networks.

### The optimal number of APFLs causing maximal occurrence of SBA

The existence of APFLs was shown to be a prerequisite for inducing SBA for 2, 3, and 4-node pulsed neural networks [2]. SRM simulations (2, 3, and 4-node small networks) indicated that only those networks containing APFLs could produce SBA. Other networks containing only negative feedback loops or double-negative positive feedback loops (referred to the section entitled "Definition of network motifs and feedback loops" for its definition) and those without any feedback loop, could not generate SBA irrespective of synaptic efficacy. We were intrigued by these observations and further investigated the relationship between APFLs and the occurrence of SBA in larger-scale networks. Figure 2 provides detailed descriptions of this relationship in both 12-node and 60-node networks. Statistical tests on these relationships were carried out for all 1,000 simulations on the same lattices, ERs (0:0.05:1) × CRs (0:0.1:1), shown in Figure 1 a-1, a-2 and Figure 1 b-1, b-2.

Figures 2 a-1 and a-2 show the correlation between two typical network behaviors, SBA and hyper-excitable activity (HEA), and the total number of 2, 3, and 4-node APFL motifs in 12-node pulsed neural networks (see Figure 2 a-1, a-2). When the number of APFL motifs increases, the mean of SBA occurrence initially increases, then reaches a peak (maximum SBA occurrence of 4.3, corresponding to four APFLs in the 12-node networks), and finally returns to zero (after 37 APFLs in the 12-node networks, see Figure 2 a-1). However, the occurrence of HEA always increased with the increase in the number of APFL motifs (see Figure 2 a-2). If the number of APFLs exceeded 37 in a 12-node network, the occurrence of 2-channel HEAs (see the section of 'Simulation protocols" for its definition) approached the maximum, $C_{2}^{12}$, which corresponds to the case in which all pairs of channels are hyper-excitable. Thus, the decreased the occurrence of SBA can be explained by the increase of HEA occurrence when APFLs are sufficiently enriched in a network. A similar result was obtained in 60-node networks (see Figures 2 b-1 and b-2). The maximum 2-channel SBA occurrence was 727, corresponding to 400 APFL motifs (2, 3, 4-node) in the 60-node networks. When the number of 2, 3, 4-node APFL motifs exceeded 900, HEA fully dominated, and all other behaviors including SBA vanished from the 60-node networks. Notably, the first points (mean = 0 and standard deviation = 0) in Figures 2 a-1 and b-1 imply that no SBA occurs when no APFL motif is present in the network. Therefore, the APFLs is necessary to trigger SBA in a pulsed neural network.

Figures 1 and 2 clearly indicate that SBA occurs significantly within an optimal range of CR and APFL number. In fact, the simulations demonstrate that the number of APFL motifs increases along with the increase of ER and CR in randomly generated synthetic networks (data not shown). Therefore, we infer that the primary factor inducing the maximal occurrence of SBA may be the formation of a suitable number of APFLs in neural networks.

### The relationship between the number of 2, 3, or 4-node APFLs and the occurrence of SBA

We also investigated the relationship between the distribution of each type of APFL and the level of SBA. In the 12-node neural networks, we found that 2-node APFLs are significantly enriched compared to 3-node or 4-node APFLs for all levels of SBA (Figure 3 a-1). Figure 3 b-1 shows the relationship between the number of 2, 3, or 4-node APFLs and the occurrence of SBA for 60-node pulsed neural networks. The number of 2-node APFLs significantly exceeds the number of 3-node or 4-node APFLs only when the occurrence of SBA exceeds a high level (1,200 events). Therefore, the number of 2-node APFLs dominates the total number of APFLs subject to a high level of SBA occurrence in both small- and large-scale networks.

How is SBA inhibited when each type of APFL motif is absent from the pulsed neural networks? Figure 3 a-2 shows the occurrence of SBA when one type or a combination of APFL motifs is not present in the 12-node networks. With the exclusion of more types of APFL motifs, both the mean and standard deviation approached zero. For example, if we take the absence of 2, 3, 4, 5, and 6-node APFLs into account, the occurrence of SBA is only 3.418 × 10^-5 ^± 0.0101 (mean frequency and standard deviation). Thus, the loss of more types of APFL motif gradually inhibits the occurrence of SBA. In 60-node networks, the observed trend slightly differs in that the exclusion of 2-node APFLs completely prohibits the occurrence of SBA (see the first point in Figure 3 b-2). This fact implies that 2-node APFLs may function dominantly in the inhibition of SBA in large-scale networks, compared with 3-node and 4-node APFLs.

### A case study of the egg-laying circuit of *C. elegans*

**Figure 4:**
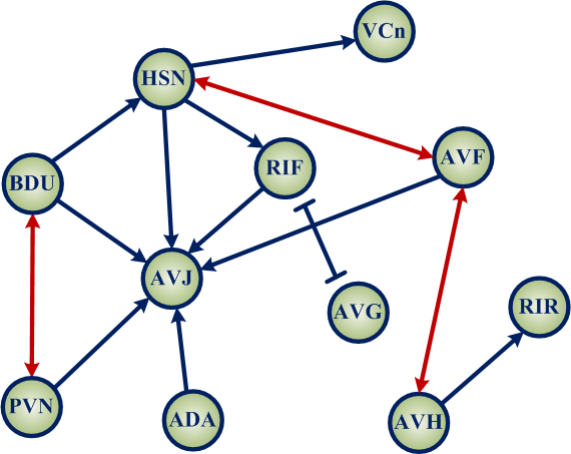
**The egg-laying circuit of *C. elegans***. Each node represents a neuron or a neuron class. The arrows represent excitatory synaptic connections and the line bars denotes inhibitory synaptic connections. In this network diagram, all outer connections are excluded for simplicity.

We investigated the egg-laying circuit of *C. elegans *composed of 11 neurons or neuron classes for a pilot study of real neural networks [32,33]. We identified that this circuit contains three two-node APFLs as indicated by the bi-directional red arrows (Figure 4). In addition, we found that the CR of this circuit is 17.3% and the ER of it is 10.5%.

We carried out simulations over two network groups for 20,000 times with different synaptic weight perturbations. One group of networks are randomly connected with any CR between 0 and 1, and the other group of networks have the same topological structure as shown in Figure 4. Two-sample *t*-test was carried out for these two groups to examine whether the real biological neural network induce higher SBAs compared to the randomly connected neural networks. Details are as follows: Let vector *x *denote the SBAs of 20,000 randomly connected networks and vector y represent the SBAs of the egg-laying neural network of *C. elegans *with the 20,000 synaptic weight perturbations. Assuming that the variances of *x *and *y *are unknown, it becomes the *Behrens-Fisher *problem [35]. The *t*-test statistic is $T=\frac{\bar{x}-ȳ}{\sqrt{s_{x}^{2}/n_{x}+s_{y}^{2}/n_{y}}}$ ~*t*(*n_x _*+ *n_y _*- 2) where *s_x _*= 4.3695 and *s_y _*= 6.9421 are the standard deviations of *x *and *y*; $\bar{x}=1.0131$ and *ȳ *= 4.9804 are the means of *x *and *y*; *n_x _*and *n_y _*are the numbers of data of *x *and *y*. The *p*-value for the null hypothesis $H_{0}:\bar{x}\geq ȳ$ is less than 0.0001 which is much less than the significance level α = 0.05. Therefore, the null hypothesis *H*_0 _should be rejected and the alternative is accepted.

## Discussion

The present study unraveled the direction of neural network development to facilitate a relatively high level of SBA. Thus, the CR range of a mature cultured neural network may represent a delicate design and not the result of random selection. In addition, such biological interpretation of the optimal CR may be further applicable to *in vivo *situations, since the distribution of cell types in *ex vivo *networks is often similar to those found *in vivo *[25,36]. Some evidence indicates that neural networks first develop toward certain connective structures and then form specific functions by adjusting their synaptic efficacies according to the external stimulus [37]. Our simulations suggest that neurons may connect with each other at a 10-30% CR to achieve the highest possibility of SBA occurrence in the early stage, and then, based on such an optimal CR range, the constructed networks further recruit and control SBA by chemically adjusting their synaptic efficacies.

We showed that our main results are quite robust to variations of network scales, network topological properties, and simulation parameters. We carried out simulations (see Simulation protocols) for a variety of neural networks with 10-300 nodes and found that the mean value of all the optimal CRs is 13% (with a standard deviation 0.0181) which lies within the evolutionarily selected range of CR (10-30%). In addition, note that the networks used for simulations in the early part (1,000 networks were constructed for each CR and ER) were based on random connections and therefore various possible topological structures were already taken into account. So, we confirmed that our results hold regardless of particular connective forms. We have also investigated the possible influences by perturbation of parameters {*τ_s_, τ_m_*, Δ_*ax*_, *τ*} (over a range from -95% to 250% perturbations of the original simulation parameters). For instance, the mean of all the optimal CRs for 10-node networks with the parameter perturbations was 18.67% (with a standard deviation 0.0183). In this way, we could also confirm that our results, the evolutionarily selected range of CR of 10-30%, still hold against the parameter perturbations.

## Conclusions

In this study, we investigated the underlying cause of the evolutionarily selected CRs of neural networks. Artificial pulsed neural network simulation has shown that an optimal CR range (10-30%) maximizes the occurrence of synchronized bursting behaviors (when ER = 0.9), which is consistent with previous *ex vivo *experimental observation, in which the CRs of cultured cortical networks consistently lie in a range of 10-30% with an ER of 80-90%.

Employing time-series data from multi-electrode array experiments, we identified some APFL motifs in cultured cortical networks of E18 Sprague-Dawley rats [2]. To further unravel the crucial role of the APFL motifs, we investigated the relationship between specific network structures (network motifs) and network behaviors in artificial pulsed neural networks. This study can readily be used to capture the fundamental dynamical characteristics of cultured neural networks. We found that the existence of APFL motifs is a necessary condition of SBA, not only for small-scale networks but also for large-scale networks. To recruit a high level of SBA, networks must have an optimal number of APFL motifs. Therefore, we infer that the formation of the appropriate number of APFLs is related to the maximal occurrence of SBA, whereas the optimal CR is only a necessary condition to achieve the required APFLs.

Furthermore, we investigated the distribution of each type of APFL motif (2, 3, or 4-node) at different SBA levels. In both 12-node and 60-node networks, the 2-node APFL motif dominated among APFL motifs at high SBA levels. More importantly, the contribution of each type of APFL motif to SBA was demonstrated by comparing the inhibitory effect of each APFL motif against SBA. For large-scale networks, the exclusion of 2-node APFLs almost fully prohibits the occurrence of SBA, implying that compared to other APFL motifs, 2-node APFL motifs may be crucial for neural networks to produce SBA.

## Methods

### Definitions of network motifs and feedback loops

A network motif is defined as an enriched sub-network pattern in complex networks that occurs more frequently than in randomized networks [38-41]. Here, this concept was extended to a more general definition. A motif refers to any sub-network with a particular structure. To relate the structure of various distinct network motifs and their dynamic behaviors, a range of different network structures can be considered, and their correlations to specific dynamic behaviors investigated. This paper focused on synchronized bursting activities.

**Figure 5:**
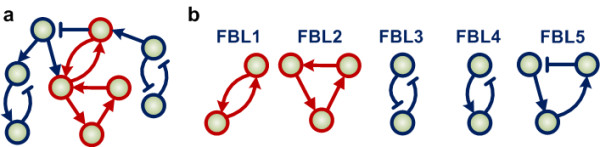
**Illustration of feedback loops and APFLs. (a) **An example network composed of five different feedback loops. An interaction between nodes is represented by arrows to denote excitatory regulation and blunt lines for inhibitory regulation. The sub-networks colored in red denote the two APFL motifs. **(b) **Five feedback loops (FBL) contained in the network shown in (a).

A feedback loop (FBL) is defined as a network motif composed of network nodes (neurons) and closed directed paths (synapses). The example network shown in Figure 5a contains five FBLs (Figure 5b). FBL1, FBL2 and FBL3 are positive feedback loops (PFLs); FBL4 and FBL5 are NFLs. The sign of a feedback loop is determined by (^-^^*1*^), where *q *is the total number of inhibitory interactions contained in the loop. Moreover, if a positive feedback loop contains only positive interactions like FBL1 and FBL2, it is referred to as an all-positive-interaction feedback loop (APFL).

### Pulsed neural networks

To infer the relationship between a type of feedback motif and its network behaviors, typical network motifs were constructed based on pulsed neural networks, and their network responses to randomly assigned initial states and simulation parameters were observed. The pulsed neural networks, also called the third generation of artificial neural networks, are based on spiking neurons, or "integrate-and-fire" neurons [22,23]. These neurons utilize recent insights from neurophysiology, specifically the use of temporal coding to pass information between neurons [11,42,43], which closely mimic realistic communication between neurons. Therefore, pulsed neural networks are commonly applied to study the properties of neural networks.

For a spiking neuron *i*, the membrane voltage can be denoted by a state variable *x_i_*. Once *x_i _*reaches a threshold δ, the neuron is fired; the moment of crossing the threshold is represented by a firing time $t_{i}^{k}$. The set of all firing times of neuron *i*, commonly called a spike train, is described as

(1) $$\Phi_{i}=\left\{t_{i}^{k}\left|u_{i}\left(t_{i}^{k}\right)\right)=\delta ;1\leq k\leq n\right\}$$

where $t_{i}^{n}$ is the most recent spike before the current time *t*. Two different processes contribute to the value of *x_i_*. The first contribution is a negative-value function $\Psi_{i}\left(t-t_{i}^{k}\right)$ indicating an immediate "reset" after each firing time in Φ_*i*_. In the biological context, Ψ*i *is used to account for neuronal refractoriness. The second contribution is inputs from pre-synaptic neurons *j *∈ *Λ_i _*where

(2) $$\Lambda_{i}=\left\{j\left|j\;\text{presynaptic to}\;i\right)\right\}$$

A pre-synaptic spike at time $t_{j}^{k}$ increases (or decreases) the state *x_i _*of post-synaptic neuron *i *for $t>t_{j}^{k}$ by summing up a weighted kernel function as $w_{ij}\varepsilon_{ij}\left(t-t_{j}^{k}\right).$ The signs can be reflected in synaptic efficacy, *w_ij_*, using *w_ij _*> 0 for excitatory synapses and *w_ij _*< 0 for inhibitory synapses. The kernel *ε_ij _*describes the response of *x_i _*due to pre-synaptic potentials at $t_{j}^{k}$, which can be viewed as a combined effect of the axonal transmission and membrane transmission properties of neurons. Therefore, the state of neuron *i *at current time *t *is given by a linear superposition of the two main previously mentioned contributions,

(3) $$x_{i}\left(t\right)=\underset{t_{i}^{k}\in \Phi_{i}}{\sum }\Psi_{i}\left(t-t_{i}^{k}\right)+\underset{j\in \Psi_{i}}{\sum }\underset{t_{j}^{k}\in \Phi_{j}}{\sum }w_{ij}\varepsilon_{ij}\left(t-t_{j}^{k}\right)$$

**Figure 6:**
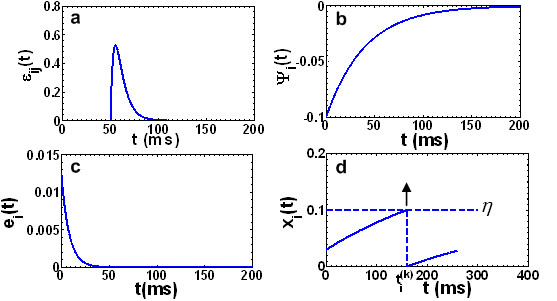
**The functions describing the dynamics of a spike neuron. (a)** The kernel ε_*ij*_(t) describing the response of *x_i_*(*t*) caused by a pre-synaptic spike at t = 0, with Δ_*ax *_= 50 *ms*, τ_s _= 3.5 *ms*, and τ_*m *_= 8 *ms*. **(b) **The function *ψ_i _*(t) reflecting refractoriness after a spike emitted at *t *= 0, with δ = 0.1 and *τ *= 40 *ms*. **(c) **The kernel *e_i_*(*t*) representing the dynamics from local current stimulation to the membrane voltage of a neuron. The time constant is identical to τ_*m *_in (a). **(d) **The membrane voltage *x_i _*(*t*) firing at time $t_{i}^{k}$ when it reaches a threshold voltage *δ *= 0.1. After firing, it is reset by the function *ψ_i_*(*t*) and then re-accumulates via the pre-synaptic spike inputs $w_{ij}\varepsilon_{ij}\left(t-t_{j}^{k}\right).$.

The models described by (1)-(3) are referred to as SRMs [22]. They, together with the connectivity topology of neural networks, form a simple mechanism of simulating biological neural networks. Frequently, noise was introduced into the SRM by adding an effect of a stochastic current $I_{i}^{noise}\left(t\right)$ to the right-hand side of (3). Then (3) can be altered to

(4) $$x_{i}\left(t\right)=\underset{t_{i}^{k}\in \Phi_{i}}{\sum }\Psi_{i}\left(t-t_{i}^{k}\right)+\underset{j\in \Psi_{i}}{\sum }\underset{t_{j}^{k}\in \Phi_{j}}{\sum }w_{ij}\varepsilon_{ij}\left(t-t_{j}^{k}\right)+\overset{\infty }{\underset{0}{\int }}e_{i}\left(s\right)I_{i}^{noise}\left(t-s\right)ds$$

where the kernel function *e*(*s*) mimics the dynamic from the local noise current stimulation to the membrane voltage of neuron *i*. As usual, several typical mathematical formulations were adopted (illustrated in Figure 6) to describe refractoriness *Ψ_i_*, post-synaptic potential ε_*ij*_, and membrane dynamics *e_i_*. For instance, let

(5) $$\varepsilon_{ij}\left(t\right)=\frac{1}{1-\left(\tau_{s}/\tau_{m}\right)}\left[\exp\left(-\frac{t-\Delta_{ax}}{\tau_{m}}\right)-\exp\left(-\frac{t-\Delta_{ax}}{\tau_{s}}\right)\right]H\left(t-\Delta_{ax}\right)$$

where τ_*s *_and τ_*m *_are time constants describing axonal transmission dynamics and membrane dynamics, respectively, and Δ_*ax *_is axonal transmission delay. *H*(*t *- Δ_*ax*_) is the Heaviside step function which vanishes for *t *≤ Δ_*ax*_, and set *t *> Δ_*ax *_equal to 1.

One typical membrane voltage reset function is

(6) $$\Psi_{i}\left(t\right)=\left\{\begin{array}{cc} -\delta \exp\left(-\frac{t}{\tau }\right), & \text{for}\;t>T_{refractory}\\ -\infty , & \text{for}\;t\leq T_{refractory} \end{array}\right)$$

where *T_refractory_*is the absolute refractory period of neuron *i*. During such a period, the neuron cannot be fired regardless of the membrane voltage. For the membrane dynamical function, the following equation can be used:

(7) $$e_{i}\left(t\right)=\frac{1}{\tau_{m}}\exp\left(-\frac{t}{\tau_{m}}\right)H\left(t\right)$$

Networks of different sizes can exhibit similar network behaviors (or dynamics) if their neurons are supplied with the same average inputs [22]. To make networks of two sizes comparable with respect to the same average input of each neuron, the networks must have weight scopes scaled by the number of network nodes. For example, take an *n*_1_-node network as a nominal case with an allowed weight scope of [-*W*_max_, *W*_max_]. Then the weight scope of an *n*_2_-node network should be assigned as $\frac{\left[-W_{\max},W_{\max}\right]}{\left(n_{2}-1\right)/\left(n_{1}-1\right)}$. In practice, the smaller network (*n*_1 _= 12) was set as the nominal network. Thus, the weight scope of the other network (*n*_2 _= 60) was scaled by the factor (60 - 1)/(12 - 1) ≈ 5.

### The typical behaviors of spike neural networks

Four typical spontaneous network behaviors appeared during the simulations: transient response activity (TRA), SBA, asynchronized bursting activities (ASBA), and HEA [44-47]. TRA is a non-sustained firing phenomenon, while both SBA and ASBA demonstrate regularly separated clusters of spikes during their full durations. The two cases can be differentiated using a synchrony index (SI) [48,49]. SI is defined based on a cross-correlation coefficient, which is often used to quantify the temporal relationship of a pair of neurons. Suppose the spike trains of the two cells in a duration of *T *seconds are denoted by *x*(*t*) and *y*(*t*) (*0 < t < T*). Their discrete-time forms *x*(*k*) and *y*(*k*) can be obtained by dividing *T *into *n *bins (*T*/bin width; *k = *1 . . . *n*) and then counting the number of spikes in each bin width. In our simulations, *T = *2 s, and bin width = 20 ms. The simulations showed that our results are quite robust with respect to arbitrary choices of *T *and bin width. The cross-correlation coefficient *r *between *x*(*k*) and *y*(*k*) is calculated as follows:

(8) $$SI=r=\frac{SS_{xy}}{\sqrt{SS_{xx}SS_{yy}}}$$

where $SS_{xx}=\overset{n}{\underset{k=1}{\sum }}x^{2}-\frac{{\left(\overset{n}{\underset{k=1}{\sum }}x\right)}^{2}}{n}$, $SS_{yy}=\overset{n}{\underset{k=1}{\sum }}y^{2}-\frac{{\left(\overset{n}{\underset{k=1}{\sum }}y\right)}^{2}}{n}$, and $SS_{xy}=\overset{n}{\underset{k=1}{\sum }}xy-\frac{\left(\overset{n}{\underset{k=1}{\sum }}x\right)\left(\overset{n}{\underset{k=1}{\sum }}y\right)}{n}$.

**Figure 7:**
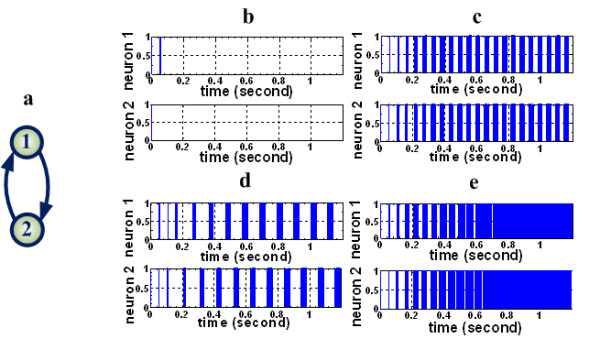
**Four possible network behaviors for a 2-node PFL motif with various synaptic efficacies**. The initial state of the network is taken as (1,1). **(a) **The scheme of a 2-node PFL motif. **(b) **Transient response activity. **(c) **Synchronized bursting activity. **(d) **Asynchronized bursting activity. **(e) **Hyper-excitable activity.

By its definition, *r *is a value in the range of [0, 1]. If *r *exceeds a threshold (0.7 was used in our simulations), the two spike trains are considered synchronized with each other. Otherwise, they are considered asynchronized. In the case of HEA, all nodes permanently fire with an interval of absolute refractory period *T_refractory_*. Notably, hyper-excited spike trains also have a high SI, which is quite similar to SBA. However, they fire as closely as possible without any perceivable intermittence during their final steady state. Figure 7 illustrates these four typical activities using a simple 2-node PFL motif. For a larger-scale network with *n *nodes, *k*-channel SBAs (2 ≤ *k *≤ *n *and *k *∈ *ℤ*) can be calculated by the same criteria. For simplicity, only 2-channel SBA was selected in this study to represent the level of *k*-channel SBAs, considering that measurements greater than 2-channel do not change the final results and major conclusions of the study. Similarly, 2-channel HEA was selected in to represent the level of *k*-channel HEAs either.

### Simulation protocols

Simulations were carried out using SRMs for the randomly connected networks where all neurons were assumed to have an identical parameter set {*τ_s_, τ_m_*, Δ_*ax*_, *τ, δ*}, and the synaptic efficacies *w_ij _*were randomly chosen from a uniform distribution [-1, 1] (see the section of 'Pulsed neural networks' for further details on SRM). For a randomly generated network with *n *neurons and *m *synaptic connections (suppose that *m_e _*denotes excitatory connections and *m-m_e _*represents inhibitory connections), the CR is defined as the percentage of the number of existing synaptic connections of the network divided by that of the fully connected network with node number *n*, i.e., $\frac{m}{n\left(n-1\right)}$. Here, only a single connection between two different neurons is allowed for simplification. Hence, the possibility of self-connection of one neuron and multiple connections between any pair of neurons are excluded. The ER is referred to as a quotient of excitatory synaptic number over the total number of synaptic connections, i.e., $\frac{m_{e}}{m}$.

To investigate the relationships among CR, ER, and the occurrence of SBA, simulations with both synaptic efficacies and network structures randomly perturbed were carried out using different combinations of CR and ER. Classification of four typical network behaviors (TRA, SBA, ASBA, and HEA) can be found in the section entitled "The typical behaviors of spike neural networks". For each ratio pair (CR, ER), 1,000 randomly connected artificial neural networks were constructed, and simulations based on these networks were carried out. For each constructed network (corresponding to one simulation), the total number of APFL motifs (2, 3, and 4-node) and the occurrences of *k*-channel SBA and HEA (2 ≤ *k *≤ *n *and *k *∈ *ℤ*) were investigated to show the correlations between APFL number and such typical behaviors. Our simulations demonstrated that the change of *k *or inclusion of more (greater than 4) node motifs had no effect on evaluating the dynamical characteristics of networks. In practice, only the 2-channel SBA and 2-channel HEA were evaluated and used to measure the levels of synchronization and hyper-excitation in networks. The correlation between the occurrence of 2-channel SBA or 2-channel HEA and network topological characteristics was obtained from the statistics of the occurrence of such behaviors with various CRs, ERs, and randomly assigned synaptic efficacies.

## Abbreviations

APFL: All-positive-interaction feedback loop; TRA: Transient response activities; SBA: Synchronized bursting activities; ASBA: Asynchronous bursting activities; HEA: Hyper-excitable activities; CR: Connective ratio; ER: Excitatory ratio; SI: Synchrony index; SRM: Spike response model.

## Competing interests

The authors declare that they have no competing interests.

## Authors' contributions

KHC designed the study, CYD performed the simulations, CYD and KHC analyzed the data, and CYD and KHC wrote the paper. All authors read and approved the final manuscript.
